# Microbial Translocation and Infectious Diseases: What Is the Link?

**DOI:** 10.1155/2012/356981

**Published:** 2012-10-04

**Authors:** Gabriella D'Ettorre, Daniel Douek, Mirko Paiardini, Giancarlo Ceccarelli, Vincenzo Vullo

**Affiliations:** ^1^Department of Public Health and Infectious Diseases, Sapienza University of Rome, 00185 Rome, Italy; ^2^Vaccine Research Center, National Institute of Allergy and Infectious Diseases, National Institutes of Health, Bethesda, MD 20892, USA; ^3^Department of Pathology & Laboratory Medicine, School of Medicine, Emory University, Atlanta, GA 30322, USA

The lumen of the gastrointestinal (GI) tract is a complex ecosystem where an enormous quantity of different bacterial species, termed the microbiota, establishes a generally symbiotic relationship with host immune system and epithelial cells. The correct interaction of the components of the GI tract permits the normal function of this ecosystem, reduces the risk of an excess of microbial translocation (MT) from the lumen of GI tract to the systemic circulation, and prevents its systemic consequences such as immune activation. From the historical observations of Berg and Garlington who defined bacterial translocation as “the passage of viable bacteria through the epithelial mucosa into the lamina propria and then to the mesenteric lymph nodes, and possibly other tissues,” this concept has been redefined several times [1, 2]. Currently, this definition has become broader and includes the passage of both viable and nonviable microbes and microbial products such as lipopolysaccharide (LPS) across an anatomically intact intestinal barrier. Recently, the concept has also attracted interest in the study of several infectious diseases such as HIV infection, hepatitis, and leishmaniasis. In the healthy host, several mechanisms are involved to prevent and/or to attenuate MT and generalized immune activation. In contrast, given the several mechanisms responsible for MT, it comes as no surprise that there are multiple infectious diseases which can be associated with MT and the consequent host response. A few years ago, a study was published that suggested a pivotal role for MT in HIV pathogenesis. The authors proposed that this phenomenon contributes to systemic immune activation in people with HIV, and thus plays a causative role in the progression of the disease [3, 4]. From these initial observations many confirmatory studies have subsequently been performed and have led to much scientific debate and reappraisal of the field of HIV disease pathogenesis. An important finding is that MT is not fully controlled by suppressive antiretroviral therapy and is associated with inefficient CD4 T-cell reconstitution. For these reasons, persistent immune activation and inflammation despite sustained antiretroviral therapy- (ART-) mediated viral suppression are able to predict subsequent mortality [5–7]. Moreover, recent studies show that MT may contribute also to the pathogenesis of non-AIDS-related morbidity, including dementia and cardiovascular diseases, and for these reasons it has emerged as a major challenge for the modern HIV treatment era [8, 9].

Notably, hepatitis B and C infections are characterized by increased levels of microbial products in the peripheral circulation. For example, during HCV infection high plasma levels of LPS were observed [10] which decreased after treatment with IFN*α*. Moreover, LPS-induced inflammation is associated with cirrhosis and predicts progression to end-stage liver disease in patients with HBV or HCV infection [11]. Despite the current data shows that MT may promote liver fibrosis either by direct interaction with Kupffer cells and hepatic stellate cells or indirectly via induction of systemic immune activation and activation-induced apoptotic cell death, further studies are clearly warranted to explore the interplay between MT and liver disease in general [12]. In addition, the HIV/HCV coinfection appears to augment the local and systemic effects of MT. In fact, increased MT in HIV-1/HCV coinfection may play an important role in the more rapid progress to fibrosis observed in coinfected patients compared to their HCV monoinfected counterparts.

In addition to what has been reported with regards to viral hepatitis and HIV infection, recent studies also show that the immunopathogenesis of visceral leishmaniasis is associated with the LPS-mediated cell activation. In fact, GI tract parasitization by Leishmania amastigotes and lymphocyte depletion could also affect the mucosal barrier and gut-associated lymphoid tissue thus predisposing to MT. Moreover, the proinflammatory response described during visceral leishmaniasis is potentiated in HIV coinfected patients and may result in a more aggressive disease progression.

Finally, the concept of MT as a driver of sepsis and multiple organ dysfunction syndrome in surgical and intensive care unit patients has emerged over the last several decades. Although the exact clinical relevance of these phenomena continues to be debated, much evidence support the hypothesis that MT is responsible for increased infectious complications in critically ill patients [13]. Furthermore, MT can be influenced not only by the severity of the patient's illness but also by the use of resuscitation procedures; for example, studies in piglets with experimental pneumonia showed increased bacterial translocation during conventional and high-PEEP ventilation [14].

The continuing debate over the role of MT in infectious diseases continues to garner much interest in the scientific world and is gradually assuming an increasingly multidisciplinary approach. From a therapeutic point of view, different interventions that aim to decrease MT are currently under evaluation. An interesting opportunity under current examination is probiotic administration; in fact, emerging studies support the concept that probiotic bacteria can provide specific benefit in HIV infection [15]. Similarly, there is evidence to support the idea that treatment with pre- and probiotics during critical illness can restore the balance of microbial communities in the gut, with beneficial effects on MT and on clinical outcome of critically ill patients.

On the basis of these findings, it is clear that MT has been recognized as an important mechanism that underlies pathogenesis in a number of different diseases. The link between MT and several infectious diseases seems to be persistent immune activation and the chronic inflammatory state. While the relative contribution of MT to the pathogenesis of different infectious diseases is likely to vary, MT *per se* seems to be a common pathway causing disease progression that is shared by different pathogens.

*Gabriella D'Ettorre* *Gabriella D'Ettorre* *Daniel Douek* *Daniel Douek* *Mirko Paiardini* *Mirko Paiardini* *Giancarlo Ceccarelli* *Giancarlo Ceccarelli* *Vincenzo Vullo* *Vincenzo Vullo*
